# Morphological and molecular characteristics of *Plasmodium juxtanucleare* in layer chicken from three districts of Yogyakarta, Indonesia

**DOI:** 10.14202/vetworld.2023.1576-1583

**Published:** 2023-08-10

**Authors:** Esti Dhamayanti, Dwi Priyowidodo, Wisnu Nurcahyo, Lintang Winantya Firdausy

**Affiliations:** 1 Department of Poultry Health and Disease Management, Veterinary Science Program, Faculty of Veterinary Medicine, Universitas Gadjah Mada, Yogyakarta, 55281, Indonesia; 2 Department of Parasitology, Faculty of Veterinary Medicine, Universitas Gadjah Mada, Yogyakarta, 55281, Indonesia

**Keywords:** avian malaria, cytochrome b gene, layer chicken, polymerase chain reaction

## Abstract

**Background and Aim:**

Blood parasite infections in poultry, such as *Plasmodium*, are a serious threat to the poultry industry due to their potential to cause economic losses. To date, there has been inadequate research on the morphological and molecular detection of the different *Plasmodium* species that infect poultry in Indonesia. Therefore, this study aimed to analyze the morphological and molecular characteristics of *Plasmodium* spp. and the several predisposing factors for *Plasmodium* infection in layer chickens from three districts of Yogyakarta, Indonesia.

**Materials and Methods:**

One hundred and five blood samples from layer chickens were collected from 13 farms located in three districts of Yogyakarta (Sleman, Bantul, and Kulon Progo) between September and November 2022. Blood samples were subjected to microscopic and polymerase chain reaction (PCR) analyses. Sequencing was performed using basic local alignment search tools to identify the nucleotide structure of cytochrome b. Phylogenetic analysis of *Plasmodium* was performed using the MEGA-X software.

**Results:**

Microscopic examination revealed that 17/105 positives (16.19%) were positive for blood parasite infection. Trophozoites, erythrocytic meronts, and microgametocytes of *Plasmodium* were found in blood samples. Based on the morphological examination, the species found in the samples was close to *Plasmodium juxtanucleare*. Polymerase chain reaction examination revealed that 21/60 samples were positive for *Plasmodium* (35%). The *Plasmodium* species identified from the sequenced samples were proven to be *P. juxtanucleare*. The *P. juxtanucleare* from Thailand was closely related to samples (99.64%–100%) with a genetic distance of 0%–1%. In addition, age, population, and cage type were not significantly associated with *Plasmodium* infection.

**Conclusion:**

Based on microscopic and PCR examinations, the Plasmodium species found in the three districts of Yogyakarta was *P. juxtanucleare*. The genetic distance between samples from the three districts of Yogyakarta was closely related (0%–1%) to *P. juxtanucleare* from Thailand and Japan. There was no correlation between *Plasmodium* infection and age, cage type, or population.

## Introduction

Avian blood parasites or haemosporidians are apicomplexan protozoa. The genera of blood parasites that infect poultry include *Leucocytozoon* spp., *Plasmodium* spp., and *Haemoproteus* spp. [1]. Blood parasite infections in layer chickens cause detrimental effects, such as stunting, decreased egg production, weight gain, and death [2]. *Plasmodium* spp. can threaten the health of poultry. Chickens infected with *Plasmodium* spp. appear anemic and lethargic due to their high parasitemia. In addition, an acute infection by *Plasmodium* spp. can cause splenomegaly in chickens. However, chickens that survive *Plasmodium* infection still experience low parasitemia even when the parasitemia level does not increase [3]. Consequently, blood parasites can become a hurdle in achieving optimum production efficiency, which can have a negative impact on poultry production required to fulfill the demand for nutritious and affordable protein sources. *Plasmodium* species found in poultry include *Plasmodium durae*, *Plasmodium gallinaceum*, *Plasmodium relictum*, and *Plasmodium juxtanucleare* [1, 4].

The susceptibility of birds to avian malaria infection can be bolstered by several predisposing factors, such as their age, host immunity, reproductive state, seasons, and temperature [2, 5]. Younger chickens tend to have more a more and prolonged parasitemia than older chickens. Age has been reported to contribute to host immunity [2]. Birds cultivated in temperate regions are prone to avian malaria, especially during hot and humid seasons such as summer and spring. These seasons allow vectors to breed and increase their activity. In addition, it promotes changes in the behavior and physiology of parasites and hosts. Changes in host physiology in terms of hormones during the breeding season also promote parasitic emergence [5]. Avian malaria infection is complex; it is difficult to separate the factors leading to host, vectors, and parasite changes. Further studies are needed to identify the factors that may promote avian malaria infection.

Studies on *Plasmodium* spp. in Indonesia are limited. Therefore, further research is needed to identify the different *Plasmodium* spp. that infects layer chickens. Molecular studies and morphological identification will help identify coinfections and cryptic diversity in species morphology that is suspected to be host-specific and define new species [3]. In addition, molecular studies on *Plasmodium* spp. will support the identification of species-specific and genetic variations among the different species. Genetic variations in pathogenic microorganisms have been predicted to cause outbreaks of poultry diseases [6].

Therefore, this study aimed to detect and identify *Plasmodium* spp. morphologically and molecularly in layer chickens from three districts of Yogyakarta, Indonesia.

## Materials and Methods

### Ethical approval

The experiments and sampling procedures were conducted according to the methods approved by the Ethics Committee of the Universitas Gadjah Mada (Approval number: 014/EC-FKH/Eks./2023).

### Study period and location

The study was conducted from September to November 2022. The samples were collected from three districts (Sleman, Bantul and Kulon Progo) of Yogyakarta. The identification processes were carried out at Laboratory of Parasitology, Faculty of Veterinary Medicine, Universitas Gadjah Mada, Yogyakarta.

### Sampling

Blood samples from 105 layer chickens were collected from 13 farms in the following three districts of Yogyakarta: Sleman (SL) (n = 41), Bantul (BA) (n = 32), and Kulon Progo (WA) (n = 32). Blood samples were collected in ethylenediaminetetraacetic acid (EDTA) tubes (StarBio^®^, China) and stored in a refrigerator. Information regarding age of the chicken, population, and cage type was collected during sampling. Chickens were divided into five age groups: Starter (0–8 weeks), grower (9–13 weeks), pre-layer (14–18 weeks), layer phase 1 (Layer-1) (19–50 weeks), and layer phase 2 (layer-2) (>51 weeks) (Table-1). Three types of cages were used, namely, battery cages composed of wood (wood), battery cages made of steel (steel), and floor pens (Table-2). The chickens were divided into three groups according to the farm population: Low (500–3000 chickens), medium (3001–5000 chickens), and high (>5000 chickens) (Table-3). The Chi-square test was used to analyze the correlation between age, population, and type of cage using statistical package for the social sciences 25 (IBM^®^, Armonk, NY, America).

**Table-1:** Microscopic and PCR examination result based on group of age from three districts in Yogyakarta.

District	Number of samples (microscopic/PCR)	Microscopic method (positive/samples)					Polymerase chain reaction, method (positive/samples)				
	
		Starter	Grower	Pre-layer	Layer-1	Layer-2	Starter	Grower	Pre-layer	Layer-1	Layer-2
SL	41/22	0/0	0/8	0/0	0/20	4/13	0/0	0/4	0/0	1/7	4/11
BA	32/19	0/0	0/0	0/0	1/20	3/12	0/0	0/0	0/0	4/9	3/10
WA	32/19	0/0	0/0	0/2	3/8	6/22	0/0	0/0	0/1	3/7	6/11

PCR=Polymerase chain reaction, SL=Sleman, BA=Bantul, WA=Kulon Progo

**Table-2:** Microscopic and PCR examination result based on type of cages from three districts in Yogyakarta.

District	Number of samples (microscopic/PCR)	Microscopic method (positive/samples)			Polymerase chain reaction, method (positive/samples)		
	
		Wood	Steel	Floor pens	Wood	Steel	Floor pens
SL	41/22	0/8	4/25	0/8	0/5	5/13	0/4
BA	32/19	3/24	1/8	0/0	4/15	3/4	0/0
WA	32/19	9/32	0/0	0/2	9/19	0/0	0/0

PCR=Polymerase chain reaction, SL=Sleman, BA=Bantul, WA=Kulon Progo

**Table-3:** Microscopic and PCR examination result based on population group from three districts in Yogyakarta.

District	Number of samples (microscopic/PCR)	Microscopic method (positive/samples)			Polymerase chain reaction, method (positive/samples)		
	
		Low	Medium	High	Low	Medium	High
SL	41/22	0/8	0/0	4/33	0/5	0/0	5/17
BA	32/19	3/24	0/0	1/8	4/15	0/0	3/4
WA	32/19	5/24	0/0	4/8			

PCR=Polymerase chain reaction, SL=Sleman, BA=Bantul, WA=Kulon Progo

### Morphological and parasitemia level examination

Blood smears were performed immediately after the blood sample collection. The blood smear was fixed with methanol (Merck^®^, Germany) until dry. Blood smears were stained with 10% Giemsa stain for approximately 30 min. Morphological examinations were performed using a microscope (OPTIKA^®^, Italy) at 1000× magnification with immersion oil (Merck^®^). Images were captured using Dp12 *microscope digital camera* (Olympus^®^, Japan) and parasite measurements were conducted using the ImageJ software (National Institutes of Health and the Laboratory for Optical and Computational Instrumentation, University of Wisconsin, USA). Parasitemia levels in blood smear samples were counted only in samples that showed positive results based on the polymerase chain reaction (PCR). Parasitemia levels were determined by counting the number of blood parasites per 2000 red blood cells. Parasitemia levels in blood samples were considered high if there were >10 blood parasites in 500 red blood cells and low if there were <10 blood parasites in 500 red blood cells [7].

### Molecular examination

Blood samples were extracted in EDTA tubes (StarBio^®^) using the wizard genomic DNA purification kit (Promega^®^, USA) with some modifications, in which the concentration of blood was 30 μL with the addition of 270 μL phosphate-buffered saline (Biogear^®^, Netherland). Polymerase chain reaction was performed using a pair of primers. The forward and reverse primers used for PCR were HaemOF (5’-CATATATTAAGAGAATTATGGAG-3’) and HaemOR (5’-ATAAAATGGTAAGAAATAC CATTC-3’), respectively [6, 8, 9]. The total volume for the PCR cycle was 25 μL, which consisted of 12.5 μL GoTaq Green Master Mix (Promega^®^, America), 5 μL DNA template, 2 μL forward primer, 2 μL reverse primer, and 3.5 μL ddH_2_O. The temperature and cycle settings were as follows: Pre-denaturation at 95°C for 5 min, denaturation at 95°C for 30 s, annealing at 50°C for 30 s, extension at 72°C for 45 s, and a final extension at 72°C for 10 min. The initial PCR was performed for 35 cycles.

Electrophoresis was performed to determine whether the PCR products were amplified successfully. To evaluate, 4.5 μL PCR products along with DNA *ladder* (GeneAid^®^, Taiwan), positive control, and negative control were run on 1.5% agarose gel that was already stained with FloroSafe DNA stain(1^st^ BASE^®^, Singapore) in an electric current of 100-volt/80-mA for 30 min. The expected PCR base length was 600 bp for the first PCR on 1.5% agarose gel, which was then electrophoresed and observed using a BluPAD UV transilluminator (Bio-Helix^®^, Taiwan). The PCR-positive samples that represented each district were then subjected to PCR sequencing with cytochrome b as the target gene using Sanger sequencing at the Integrated Research and Testing Laboratory, Universitas Gadjah Mada.

### Phylogenetic analysis

Molecular data obtained from the PCR sequencing of selected positive PCR amplification products were processed using basic local alignment search tools (BLAST) (Bethesda, Maryland, USA) (www.ncbi.nlm.nih.gov/BLAST) and MEGA-X (Pennsylvania State University, Pennsylvania, USA) (https://www.megasoftware.net/). The neighbor-joining method with the Kimura 2 parameter distance matrix was used to examine the genetic relationship of blood parasites from this research sequence with published sequences.

## Results

### Morphological analysis of the *Plasmodium* spp.

**Figure-1:**
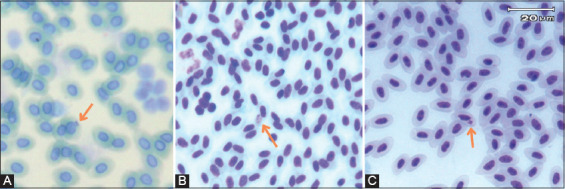
Trophozoites phase of *Plasmodium juxtanucleare* (A); Microgametocyte (B); Erythrocytic meront (C).

Based on blood smear examination with Giemsa staining, 16.2% (17/105) of the blood smear samples were positive. The early trophozoite phase of *Plasmodium* spp. was the dominant phase found in the blood samples. In addition, other *Plasmodium* stages, such as erythrocytic meront and microgametocyte, were detected in the samples (Figure-1).

*Plasmodium* trophozoites found on the samples were more oval or round and adhered to the nuclei of the host red blood cells. *Plasmodium* erythrocytic meronts were located close to the erythrocyte nucleus and were oval in shape, with pale and sparse cytoplasm. *Plasmodium* microgametocytes were also located near the erythrocyte nucleus and were oval, with pale and vacuolar cytoplasm. The length and width of microgametocytes of *Plasmodium* were 4.1 μm and 2.7 μm, respectively. The morphology of *P. juxtanucleare* is illustrated in Figure-1.

### Molecular identification to determine *Plasmodium* species

**Figure-2:**
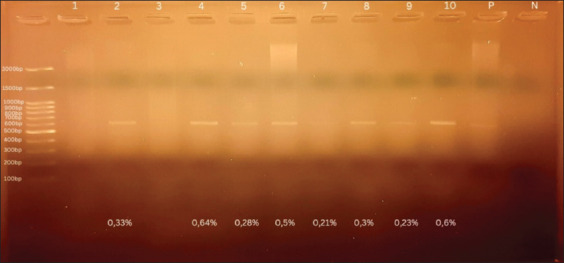
Results of PCR on agarose gel with the percentage of parasitemia from each positive band. PCR negative results (Lanes 1 and 3); PCR-positive results (Lanes 2 and 4—10); positive control (P) from sample proved by sequencing; negative control (N).

Molecular identification using PCR revealed 21 of the 60 samples as positive (35%). Positive samples detected by PCR showed a band at 600 bp on 1.5% agarose gel (Figure-2). Nine sequenced samples from SL, BA, and WA were closely related to *P. juxtanucleare*, according to BLAST searches. Samples along with the accession numbers from SL 7: OR039276, SL 8: OR050812, and SL 9: OR050813, BA 6: OR050814 and BA 24: OR050815, and WA 17: OR050817 and WA 30: OR050818 were closely related (98.81%–100%) to *P. juxtanucleare* (LC713397.1), (KU248845.1), and (NC_008279.1) from Thailand and Japan, respectively. However, two sequenced PCR samples from WA along with the accession numbers (WA 10: OR050816 and WA 24: OR050819) were closely related (99.64%–100%) to *P. juxtanucleare* (LC713398.1) from Thailand.

**Figure-3:**
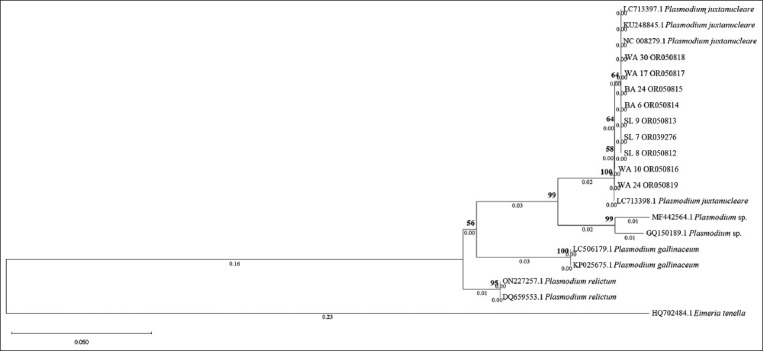
Phylogenetic tree gene cytochrome b of samples, *Plasmodium* spp., *Plasmodium relictum*, *Plasmodium juxtanucleare*, and *Plasmodium gallinaceum* based on nucleotide sequence using Neighbor-Joining method along with Kimura 2 parameter. Sleman (SL); Bantul (BA); Kulon Progo (WA).

Nine sequenced samples from SL, WA, and BA also showed low genetic distance, which was only 0%–1% (Table-4) based on sequence alignment using MEGA-X. Samples from WA 10 and WA 24 and *P. juxtanucleare* (LC713398.1) differed by one nucleotide base located at 299 bp, which contained thymine (T) instead of cystine (C) (Table-5). Therefore, samples from WA 10 and WA 24 and *P. juxtanucleare* (LC713398.1) were placed in the same group, whereas rest of the samples from SL, WA, and BA and *P. juxtanucleare* LC713397.1, KU248845.1, and NC_008279.1 formed the other group in the same clade with 100% bootstrap value (Figure-3). The majority of sequenced samples from three districts in Yogyakarta showed a close genetic distance to *P. juxtanucleare* from Thailand (LC713397.1), (KU248845.1), and Japan (NC_0082791), except WA 10 and WA 24, which were similar to *P. juxtanucleare* (LC713398.1) from Thailand.

**Table-4:** Genetic distance between samples and *Plasmodium juxtanucleare* from Thailand and Japan.

	SL 7	SL 8	SL 9	BA 6	BA 24	WA 10	WA 17	WA 24	WA 30	NC_008279.1	LC713398.1	LC713398.1	KU248845.1
SL 7 OR039276													
SL 8 OR050812	0												
SL 9 OR050813	0	0											
BA 6 OR050814	0	0	0										
BA 24 OR050815	0	0	0	0									
WA 10 OR050816	1	1	1	1	1								
WA 17 OR050817	0	0	0	0	0	1							
WA 24 OR050819	1	1	1	1	1	0	1						
WA 30 OR050818	0	0	0	0	0	1	0	1					
NC_008279.1 *P. juxtanucleare* (Japan)	0	0	0	0	0	1	0	1	0				
LC713398.1 *P. juxtanucleare* (Thailand)	1	1	1	1	1	0	1	0	1	1			
LC713397.1 *P. juxtanucleare* (Thailand)	0	0	0	0	0	1	0	1	0	0	1		
KU248845.1 *P. juxtanucleare* (Thailand)	0	0	0	0	0	1	0	1	0	0	1	0	

**Tabel-5:** Nucleotide sequences of samples and *Plasmodium juxtanucleare* from Thailand and Japan.

Samples/Accession Number	Nucleotide Position								

	290	293	296	299	302	305	308	311	314
SL 7 OR039276	A	C	A	C	A	C	A	T	T
SL 8 OR050812	A	C	A	C	A	C	A	T	T
SL 9 OR050813	A	C	A	C	A	C	A	T	T
BA 6 OR050814	A	C	A	C	A	C	A	T	T
BA 24 OR050815	A	C	A	C	A	C	A	T	T
WA 10 OR050816	A	C	A	T	A	C	A	T	T
WA 17 OR050817	A	C	A	C	A	C	A	T	T
WA 24 OR050819	A	C	A	T	A	C	A	T	T
WA 30 OR050818	A	C	A	C	A	C	A	T	T
NC_008279.1 *P. juxtanucleare* (Japan)	A	C	A	C	A	C	A	T	T
LC713398.1 *P. juxtanucleare* (Thailand)	A	C	A	T	A	C	A	T	T
LC713397.1 *P. juxtanucleare* (Thailand)	A	C	A	C	A	C	A	T	T
KU248845.1 *P. juxtanucleare* (Thailand)	A	C	A	C	A	C	A	T	T

**Figure-4:**
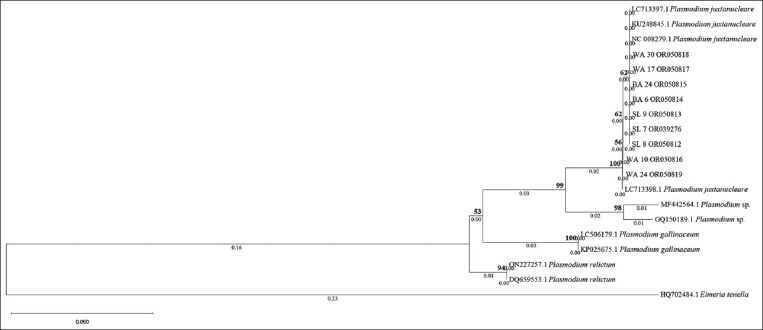
Phylogenetic tree gene cytochrome b of samples, *Plasmodium* spp., *Plasmodium relictum, Plasmodium juxtanucleare*, and *Plasmodium gallinaceum* based on protein sequence using Neighbor-Joining method along with Kimura 2 parameter. Sleman (SL); Bantul (BA); Kulon Progo (WA).

The protein sequences of WA 10 and WA 24 also showed a difference in one amino acid position (Table-6) with leucine (L) instead of serine (S). An amino acid position difference was also observed in *P. juxtanucleare* (LC713398.1), with L instead of S in the same amino acid position as that of WA 10 and WA 24. There were no changes in the phylogenetic tree position based on the protein sequence (Figure-4).

**Table-6:** Amino acid position of samples and *Plasmodium juxtanucleare* from Thailand and Japan.

Samples/Accession Number	Amino Acid Position								

	91	94	97	100	103	106	109	112	115
SL 7 OR039276	F	Y	Y	S	Y	V	H	F	L
SL 8 OR050812	F	Y	Y	S	Y	V	H	F	L
SL 9 OR050813	F	Y	Y	S	Y	V	H	F	L
BA 6 OR050814	F	Y	Y	S	Y	V	H	F	L
BA 24 OR050815	F	Y	Y	S	Y	V	H	F	L
WA 10 OR050816	F	Y	Y	L	Y	V	H	F	L
WA 17 OR050817	F	Y	Y	S	Y	V	H	F	L
WA 24 OR050819	F	Y	Y	L	Y	V	H	F	L
WA 30 OR050818	F	Y	Y	S	Y	V	H	F	L
NC_008279.1 *P. juxtanucleare* (Japan)	F	Y	Y	S	Y	V	H	F	L
LC713398.1 *P. juxtanucleare* (Thailand)	F	Y	Y	L	Y	V	H	F	L
LC713397.1 *P. juxtanucleare* (Thailand)	F	Y	Y	S	Y	V	H	F	L
KU248845.1 *P. juxtanucleare* (Thailand)	F	Y	Y	S	Y	V	H	F	L

### Predisposition factors: Age, cage type, and population

The age of the chicken ranged from 9 to 84 weeks. The population of layer chickens in each farm was 500–53,000. The cages used in layer chicken farms were battery cages made of wood, steel, and non-battery cages. Most farms in the districts of Yogyakarta use wooden battery cages. Based on the age of the chickens, nine samples from layer-1 and 12 samples from layer-2 were detected as positive by PCR, whereas four samples from layer-1 and 13 samples from layer-2 were detected as positive by microscopic examination (Table-1). Based on the type of cage, 14 chickens were kept in wood battery cages and seven chickens were positive for *Plasmodium* infection using PCR, whereas 12 chickens kept in wood battery cages and five in steel battery cages were detected as positive by microscopic examination (Table-2). Moreover, based on population data, *Plasmodium* infection was detected in ten chickens from low-population farms and 11 chickens from high-population farms using the PCR method, while nine samples from high-population farms and eight samples from low-population farms were detected as positive by the microscopic method (Table-3). The Chi-square significance value for age of the chicken according to microscopic analysis was 0.11, whereas the Chi-square significance value for PCR examination was 0.28. The Chi-square significance value for the type of cage based on microscopic examination was 0.39, whereas that based on PCR examination was 0.19. In addition, the Chi-square significance value of the microscopic examination based on the chicken population was 0.57, whereas that of the PCR analysis was 0.23. Thus, according to the Chi-square analysis, there was no correlation between plasmodiasis and the age of the chickens, the type of cages, and the population of layer chickens in farms (p < 0.05).

## Discussion

Based on morphological characterization, the *Plasmodium* spp. found in the samples of the present study had the same characteristics as *P. juxtanucleare*. This finding is supported by studies showing similar morphological characteristics [7, 10, 11]. *Plasmodium juxtanucleare* trophozoites, meronts, and gametocytes tend to attach to the nuclei of host erythrocytes. Morphological examination using a microscope to determine the species of *Plasmodium* or any blood parasite is challenging and requires ample skill [10]. In the present study, the most abundant stages among the positive samples were *P. juxtanucleare* trophozoites, which indicate that the chickens may have been in the early stages of *P. juxtanucleare* infection. These results are corroborated by the findings of Tattiyapong *et al*. [10] and Silveria *et al*. [12], and are supported by PCR results.

Samples that underwent PCR showed more positive results than those that underwent a microscopic examination. Polymerase chain reaction has higher sensitivity than the microscopic examination. Silveria *et al*. [12], detected approximately twice as many positive results for *P. juxtanucleare* using PCR. The thickness of the band on the agarose gel for each sample indicated the parasitemia level (Figure-2). As shown in Figure-2, the lowest parasitemia level was 0.21%, represented by the thinnest band, whereas the highest parasitemia level among the positive samples was 0.64%, represented by the thickest band. Bensch *et al*. [13], showed a correlation between the thickness of the band on agarose gel and the rate of erythrocyte infection. The thinnest band was observed at 0.04%, whereas the thickest was observed at 1.93%.

Nucleotide differences between T and C in WA 10 and WA 24 may indicate a transversion in which a mutation of the nucleotide base has the same ring (pyrimidine to pyrimidine or purine to purine) [14]. The change in one nucleotide base in WA 10 and WA 24 influenced its protein sequence, which differed between L and S. Changes or mutations in protein sequences affect protein function [15]. A single mutation in codon 268 *cytb* of *P. falciparum* can cause resistance to atovaquone [16]. The nucleotide and protein sequences of WA 10 and WA 24 may be influenced by drug exposure targeting the mitochondrial electron transport chain (ETC). Disruption of ETC interferes with metabolic processes, such as *de novo* pyrimidine synthesis, which is important for nucleic acid replication [17]. However, protein mutations generally occur naturally and do not evoke changes in protein function. This phenomenon is known as protein robustness [18, 19]. Further research is needed to elucidate the causes of nucleotide and protein sequence changes in *P. juxtanucleare*. The phylogenetic tree based on the nucleotide and protein sequences in this study was identical.

Younger chickens are more susceptible to parasitic infection and have higher parasitemia levels, prolonged parasitemia, and higher mortality rates than older chickens [2]. The chickens infected with *P. juxtanucleare* in this study were older. Therefore, the clinical symptoms were mild, such as paleness of the comb, wattle, and lethargy. The clinical symptoms of *P. juxtanucleare* infection can be moderate, mild, or asymptomatic [20, 21], and chickens infected with *P. juxtanucleare* may exhibit lethargy, white diarrhea, and convulsions [10]. The adaptation of chicken immunity to *P. juxtanuclear* infection may influence the severity of infection [20]. Chicken immunity can also be affected by cage type. Chickens in battery cages showed lower immunity due to chronic stress than chickens in enriched cages. On the other hand, chickens kept in enriched cages and free-range tend to have a higher chance of being infected by the disease [22, 23]. No previous research has been conducted to determine the relationship between farm chicken populations and avian malaria. This study showed no relationship between the age of the chickens, type of cage, and population, which may be due to the insufficient sample size.

## Conclusion

Based on microscopic and PCR examinations, the *Plasmodium* species found in layer chickens from the three districts of Yogyakarta, Indonesia, was *P. juxtanucleare*, which had a low genetic distance from *P. juxtanucleare* from Thailand and Japan (0%–1%). There was no correlation between *Plasmodium* infection and age, type of cage, and population of layer chickens.

## Authors’ Contributions

ED, DP, and WN: Conceptualization, methodology, and data curation. ED: Resources and funding. ED, DP, WN, and LWF: Project administration, supervision, writing-review, and editing. ED and LWF: Formal analysis and visualization. ED, DP, WN, and LWF: Investigation and validation. All authors have read, reviewed, and approved the final manuscript.
